# Targeting Chronic Biofilm Infections With Patient-derived Phages: An In Vitro and Ex Vivo Proof-of-concept Study in Patients With Left Ventricular Assist Devices

**DOI:** 10.1093/ofid/ofaf158

**Published:** 2025-03-20

**Authors:** Melissa Pitton, Luca G Valente, Simone Oberhaensli, Bülent Gözel, Stephan M Jakob, Parham Sendi, Monika Fürholz, David R Cameron, Yok-Ai Que

**Affiliations:** Department of Intensive Care Medicine, Inselspital, Bern University Hospital, University of Bern, Bern, Switzerland; Graduate School for Cellular and Biomedical Sciences (GCB), University of Bern, Bern, Switzerland; Department of Intensive Care Medicine, Inselspital, Bern University Hospital, University of Bern, Bern, Switzerland; Graduate School for Cellular and Biomedical Sciences (GCB), University of Bern, Bern, Switzerland; Institute for Infectious Diseases, University of Bern, Bern, Switzerland; Interfaculty Bioinformatics Unit, University of Bern, Bern, Switzerland; SIB Swiss Institute of Bioinformatics, Lausanne, Switzerland; Department of Intensive Care Medicine, Inselspital, Bern University Hospital, University of Bern, Bern, Switzerland; Department for BioMedical Research, University of Bern, Bern, Switzerland; Department of Intensive Care Medicine, Inselspital, Bern University Hospital, University of Bern, Bern, Switzerland; Institute for Infectious Diseases, University of Bern, Bern, Switzerland; Department of Cardiology, Inselspital, Bern University Hospital, University of Bern, Bern, Switzerland; Department of Intensive Care Medicine, Inselspital, Bern University Hospital, University of Bern, Bern, Switzerland; Department of Intensive Care Medicine, Inselspital, Bern University Hospital, University of Bern, Bern, Switzerland; Department for BioMedical Research, University of Bern, Bern, Switzerland

**Keywords:** biofilms associated infections, left ventricular assist devices, medical microbiology, multidrug resistant *Staphylococcus epidermidis*, personalized phage therapy

## Abstract

**Background:**

Phage therapy is being reconsidered as a valuable approach to combat antimicrobial resistance. We recently established a personalized phage therapy pipeline in healthy volunteers, where therapeutic phages were isolated from individuals' skin microbiota. In this study, we aim to validate this pipeline in end-stage heart failure patients supported by left ventricular assist devices (LVADs), focusing on phages targeting *Staphylococcus epidermidis*, a common pathogen responsible for LVAD infections.

**Methods:**

Over a 2.5-year period, 45 LVAD patients were consistently sampled at their driveline exit sites and foreheads. *S epidermidis* strains from patients' foreheads were used to amplify patient-specific phages. Newly isolated phages were characterized and tested against *S epidermidis* isolates (n = 42) from the patient cohort. The virulent phage vB_SepS_BE22, isolated from a patient with a driveline infection, was further tested for its bactericidal activity against *S epidermidis* biofilms ex vivo with rifampicin on driveline biofilms.

**Results:**

*S epidermidis* was detected in 32 patients, 3 of whom had driveline infections. Phages were isolated from 8 patients, 6 of which were unique and exhibited narrow host ranges, infecting 19%–52% of *S epidermidis* strains. vB_SepS_BE22, isolated from patient ID25's microbiota, was the only phage that specifically killed *S epidermidis* clones linked to a patient's infection. vB_SepS_BE22 also reduced bacterial loads in exponential and stationary phase cultures, as well as in biofilms on drivelines when combined with rifampicin.

**Conclusions:**

This study validated a personalized phage therapy approach, where phages from a patient's own microbiota can be used in chronic infection settings as therapeutic agents.

## Teaser

This study demonstrates the use of personalized phage therapy to target *Staphylococcus epidermidis* infections in patients with left ventricular assist devices. It highlights the potential of patient-specific phages to reduce biofilm-associated infections, especially in combination with antibiotics like rifampicin.

The misuse of antibiotics has expedited the antimicrobial resistance crisis and contributed to an increasing number of chronic, hard-to-eradicate bacterial infections [1, 2]. In parallel, medical progress has promoted the widespread use of indwelling medical devices and implants, such as catheters, orthopedic prostheses, mechanical heart valves, and heart-failure support devices that all provide favorable niches for opportunistic bacteria to cause infections, often in association with biofilms [3–5].

*Staphylococcus epidermidis* has emerged as a clinically relevant healthcare-associated opportunistic pathogen and is one of the leading causes of infections on indwelling medical devices and implants [6–8]. *S epidermidis* infections are typically hard to eradicate [9]. On one hand, *S epidermidis* has developed resistance to various antibiotic classes as confirmed by high antibiotic resistance rates reported among healthcare-associated and nosocomial isolates [10]. On the other, *S epidermidis* has an extraordinary capacity to form robust biofilms [11, 12], adhering to diverse surfaces and encasing itself in extracellular matrices that limit the action of antibiotics [13, 14]. There is an urgent need for alternative strategies that address each of these issues.

One treatment option for *S epidermidis* infections involves the use of bacterial viruses (bacteriophages/phages), referred to as phage therapy. The very nature of phages make them attractive for the treatment of biofilm infections caused by antibiotic resistant staphylococci [15, 16]; the mechanism of killing is distinct from traditional antibiotic classes decreasing the likelihood of phage cross resistance in antibiotic-resistant strains [16], and some phages have the capacity to degrade biofilms [17]. Yet, there are several barriers to the immediate application of phage therapy for the management of *S epidermidis* biofilm infections. When compared to phages infecting closely related species such as *Staphylococcus aureus* [18, 19], phages targeting *S epidermidis* often have very narrow host ranges [20], which preclude their use as a “one-size-fits-all” approach [21]. In addition, *S epidermidis* virulent phages available for therapy are scarce despite efforts to isolate them from various environmental sources [22, 23].

We recently proposed a phage hunting pipeline using the human skin microbiota as a source for isolation of *S epidermidis* phages. We selected this specific reservoir because phages require a susceptible host for propagation, and human skin harbors *S epidermidis*; therefore, making it a suitable source for phage isolation. We evaluated this approach in healthy individuals and isolated novel phages directed against *. epidermidis,* 1 of which was shown to kill *S epidermidis* embedded in biofilms [20]. The goal of the present study was to validate the feasibility of this approach in patients prone to hard-to-treat *S epidermidis* infections. As proof-of-concept, we focused on patients with end-stage heart failure placed on left ventricular assist device (LVAD) support, as these patients often develop chronic, hard-to-treat driveline infections due to *S epidermidis* [24–28]. We investigated whether phages can be isolated from the patient's skin microbiota using the patients' specific *S epidermidis* as the propagating organism, and subsequently tested the capacity of the phages to disrupt *S epidermidis* biofilms formed on explanted driveline material.

## MATERIALS AND METHODS

### Study Design and Patient Population

This prospective, single-center, longitudinal, observational study approved by the ethical committee of the Canton Bern (#2019-00768) and conducted at the Bern University Hospital, Switzerland, between June 2019 and December 2021, has previously been described in detail [29]. All patients aged >18 years with LVAD and with at least 1 regular follow-up visit at the outpatient consultation of the Department of Cardiology of the Bern University Hospital were eligible and included after written informed consent.

### Isolation of *S epidermidis* From Patients

*S epidermidis* strains were isolated from the driveline exit sites using sterile swabs, and from patients' foreheads using dry sterile tissues. Swabs were streaked out onto Columbia Sheep Blood Agar, Columbia Sheep Blood Agar–colistin/nalidixic acid, and MacConkey agar plates before incubation at 37 °C for 24 hours. Tissues were immersed in 15 mL of sodium-magnesium buffer (100 mM NaCl, 8 mM MgSO_4_, tris-HCl [30]) and vortexed at maximum speed for 5 minutes. Aliquots (100 μL) were spread onto mannitol salt agar plates and incubated overnight at 37 °C. Colonies not showing mannitol fermentation were subcultured onto fresh mannitol salt agar plates and incubated at 37 °C for an additional 24 hours. Microflex LT Matrix-Assisted Laser Desorption/Ionization Biotyper (Bruker, Billerica, Massachusetts, USA) was used to confirm the presence of *S epidermidis* according to the instructions of the manufacturer.

### Bacteriophage Isolation

Phage isolation was performed as described previously [20] from the same tissue sample used to isolate *S epidermidis* from the forehead. *S epidermidis* isolated from each respective patient's forehead was used as strain for the first propagation steps.

### Genome Sequencing and Analysis

Phage DNA was extracted using the Phage DNA Isolation Kit (Norgen Biotek Corp., Canada) according to the manufacturer's instructions. For phage genome sequencing, libraries were generated using Nextera DNA flex Library Prep Kits (Illumina, San Diego, California, United States) and then sequenced using the Illumina MiSeq platform (2 × 150 bp). Raw reads were assembled using SPAdes (v3.15.2) in isolate mode [31]. Genomes were annotated using Pharokka (v1.2.0) [32]. Phage genomes comparisons were performed using BLASTn [33]. Genome-based phylogeny was inferred using VICTOR [34], and the tree was modified using Interactive Tree Of Life [35]. Phage genomes were screened for virulence genes using VirulenceFinder 2.0 [36]. Circular representations of genomes were generated with Proksee (https://proksee.ca/).

Bacterial genomic DNA was extracted using a DNeasy Ultraclean Microbial kit (Qiagen, Hilden, Germany) then sequenced using a PacBio Sequel platform. Reads were assembled using Flye (v2.8.3) [37]. Genome sequences were annotated using the NCBI Prokaryotic Genome Annotation Pipeline [38]. Core gene alignment was created using Roary [39], and the phylogenetic tree was generated using RAxML-GTR GAMMA (Rapid hill-climbing algorithm) [40]. Phage and bacterial genomes were deposited at DDBJ/ENA/GenBank under the accession numbers listed in Table 1 and in Supplementary Table 1, respectively.

**Table 1.** Characteristics of Phages Isolated From LVAD Patients

Name	Family	Genus	Length (bp)	GC%	CDS	Patient ID	Lifestyle	Accession Number	Homology With *S epidermidis* Phages
vB_SepS_BE20 Figure S3A	*Siphoviridae*	*Phietavirus*	43 521	35	82	1, 44, 46	Temperate	OQ355699	vB_SepS_459, vB_SepS_27, vB_SepiS-phiIPLA7 [41, 42].
vB_SepS_BE21 Figure S3B	*Siphoviridae*	*Phietavirus*	43 563	35	75	24	Temperate	OQ355700	vB_SepS_BE01, vB_SepS_459, vB_SepS_27 [20]
vB_SepS_BE28 Figure S3C	*Siphoviridae*	*Phietavirus*	42 841	35	74	14	Temperate	OQ355704	vB_SepS_459, vB_SepS_27, IME1348_01
vB_SepS_BE22 Figure S3D	*Siphoviridae*	*Sextaecvirus*	92 847	29	167	25	Virulent	OQ355701	phage 6ec, vB_SepS_BE02, vB_SepS_SEP9 [20, 43, 44]
vB_SepM_BE24 Figure S3E	*Herellenviridae*	*Sepunavirus*	140 570	28	167	32	Virulent	OQ355702	vB_SepM_BE06, phage Terranova, phage Quidividi [45]
vB_SepM_BE25 Figure S3F	*Herellenviridae*	*Sepunavirus*	140 292	28	240	16	Virulent	OQ355703	phiIPLA-C1C, phage Twillingate, Phage Terranova [23, 45]

Abbreviations: bp, base pairs; CDS, coding sequence; LVAD, left ventricular assist device.

### Host Range Characterization

Phage host ranges were determined using a standard spot test assay on double-layer agar [46] using a panel of 42 fully sequenced *S epidermidis* isolates collected from the patient cohort (Supplementary Table 1). Phages were primarily isolated and propagated using *S epidermidis* strains from patients' foreheads, then further amplification steps were performed using *S epidermidis* strain F12 [22], which is a prophage-free strain, and used as reference for efficiencies of plating determination.

### Time-kill Assays in Planktonic Cultures

Two *S epidermidis* strains were used for these experiments; strain 25DSE01 was isolated from the infected driveline exit site of patient ID25 harboring phage vB_SepS_BE22, whereas strain 1FSE05 was isolated from patient ID1. *S epidermidis* overnight cultures were diluted 1:100 and incubated in Trypticase Soy Broth (TSB; BD, Franklin Lakes, New Jersey, USA) at 37 °C with shaking (200 rpm). Bacterial cell cultures at late logarithmic (∼8 × 10^7^ colony-forming unit [CFU]/mL) or stationary (∼2 × 10^9^ CFU/mL) phase of growth were exposed to phage vB_SepS_BE22 (∼2 × 10^7^ PFU), rifampicin (at the minimal inhibitory concentration [MIC]; ie, 0.008 µg/mL), or a combination of both, and further incubated at 37 °C for 24 hours. Bacterial viability was determined at 0, 4, and 24 hours after treatment (limit of detection 10^2^ CFU/mL). To prevent phage and/or rifampicin carryover, samples were washed twice by centrifugation at 5000*g* for 10 minutes. Emergence of phage resistance was tested using a cross-streaking assay as described elsewhere [19], and rifampicin resistance was tested by assessing the MIC using the standard broth microdilution assay [47].

### Biofilm Assay Using 96-well Microtiter Plates

Biofilms were produced in 96-well microtiter plates as described by O’Toole [48], with minor modifications. Briefly, overnight cultures of *S epidermidis* 25DSE01 were diluted 1:100 in TSB and supplemented with 0.25% glucose (125 µL per well). Following incubation at 37 °C for 24 hours, planktonic cells were removed by washing with sterile water. Phages diluted in TSB were added (∼2 × 10^7^ PFU, 150 µL per well) and the plates were incubated for 4 hours at 37 °C. Sodium-magnesium buffer was used for untreated controls. Then, biofilms were washed and stained with crystal violet. To dissolve crystal violet, acetic acid (30%) was added to each well and the optical density was measured at 550 nm (MiniMax 300 Imaging Cytometer, Molecular Devices, San Jose, California, United States). The experiment was performed in triplicates on 2 different days (n = 6). Statistical comparisons between cultures treated with and without phages were conducted using an unpaired *t* test, with a *P* value ≤ .05 considered statistically significant.

### Ex Vivo Biofilm Assay on Explanted Driveline Sections

Biofilms were generated on 0.5-cm sections of explanted LVAD drivelines, which had been cleaned and sterilized. Driveline sections in 24-well plates were immersed in 2 mL of TSB supplemented with 0.25% glucose containing ∼2 × 10^7^ CFU/mL of bacteria before incubation at 37 °C for 24 hours. Following incubation, sections were rinsed 3 times in phosphate-buffered saline and transferred to a well containing 2 mL of either phages (∼2 × 10^7^ PFU), rifampicin (0.008 µg/mL), a combination phages/rifampicin, or TSB (untreated control); and then incubated for 24 hours at 37 °C. After incubation, each driveline section was washed 3 times in phosphate-buffered saline and transferred to a tube containing 1 mL of NaCl (0.9%). Biofilm detachment was achieved by combining sonication (10 minutes) and vortexing (2 minutes). To avoid the carryover of treatments, samples (200 µL) were washed twice by centrifugation (5000*g*, 10 minutes) using NaCl (0.9%). CFU were enumerated on TSB agar. Six driveline sections were analyzed per condition. Statistical comparison was performed using 1-way analysis of variance test with Tukey's multiple comparisons. Data were considered significantly different when *P* ≤ .05.

## RESULTS

### Study Design

**Figure 1.**
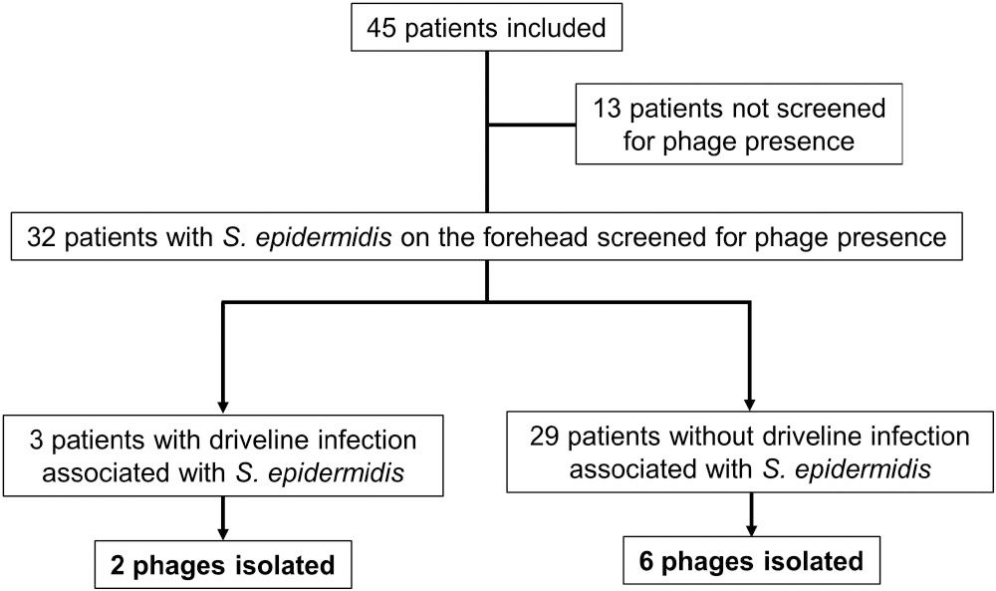
Study flowchart. Thirteen patients were excluded either because of the absence of *S epidermidis*, which made phage screening unfeasible as there was no appropriate host bacteria to test our hypothesis (n = 2), or because of transfer to another hospital (n = 1), late enrollment (n = 1), heart transplantation (n = 2), or death (n = 7).

Forty-five patients with end-stage heart failure on LVAD support were regularly swabbed at the driveline exit site over a 2.5-year time period [29]. Of 32 patients screened for phages using the *S epidermidis* strains isolated from the patients' own forehead as propagating strains [20] (Supplementary Fig. 1), 18 developed driveline infections, which were associated with *S epidermidis* in 3 instances (Figure 1, Supplementary Table 2). In total, 8 *S epidermidis* phages were stably propagated, phenotypically characterized, and genome sequenced (Table 1). In 1 instance, a patient was screened twice at independent visits, and plaques were observed in both assays, confirming the reproducibility of the method.

### Characterization of the Newly Isolated Phages

Genome and electron microscopy analyses identified 3 unique temperate and 3 unique virulent phages (Table 1, Supplementary Fig. 2), classified into the *Siphoviridae* (n = 4) and *Herellenviridae* (n = 2) families. Notably, 3 patients shared the same siphovirus (vB_SepS_BE26 and vB_SepS_BE27 were fully identical to vB_SepS_BE20). Three of the unique siphoviruses (vB_SepS_BE20, vB_SepS_BE21, and vB_SepS_BE28) belong to the *Phietavirus* genus, whereas the fourth (vB_SepS_BE22) belongs to the *Sextaecviruses* genus (Table 1, Supplementary Fig. 3). Interestingly, vB_SepS_BE22 lacks the complete set of lysogeny-related genes typically found in *sextaecviruses*, suggesting a virulent behavior. The remaining 2 unique phages, vB_SepM_BE24 (Table 1, Supplementary Fig. 3E) and vB_SepM_BE25 (Table 1, Supplementary Fig. 3F), belong to the *Herellenviridae* family.

### Isolated Phages Exhibit a Narrow Host Range

**Figure 2.**
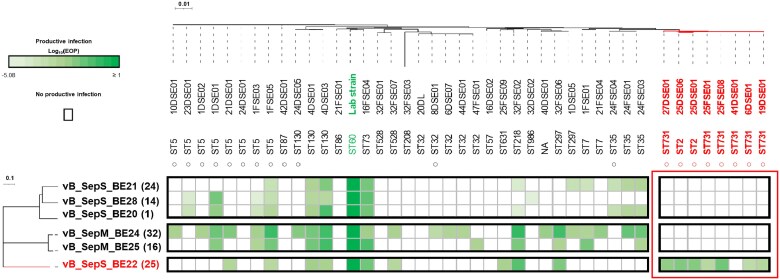
Host range characterization of phages isolated from patients with left ventricular assist device. Phages (vertically) and *S epidermidis* isolates (horizontally) have been ordered based on phylogeny. For phages, the patient ID has been added in brackets after phage names, whereas *S epidermidis* strains have been named after patient ID (ie, strain 10DSE01 has been isolated from patient ID10). Sequence type (ST) has been inferred using multilocus sequence typing. The 6 unique phages identified in the study were tested for their ability to infect a representative panel of 42 *S epidermidis* isolates. A productive phage infection was defined as the presence of plaques and expressed in efficiency of plating (EOP) compared to the *S epidermidis* F12 (laboratory reference strain used also for phage amplification (shown in green). Methicillin-resistant *S epidermidis* (MRSE) isolates harboring the *mecA* gene are indicated with a circle (○). The ability of vB_SepS_BE22 to infect MRSE ST2/731 clones has been highlighted in red.

Newly isolated phages were tested against a panel of 42 fully sequenced *S epidermidis* isolates collected from the patient cohort, and each displayed a narrow host range (19%–52%) (Figure 2). The panel of *S epidermidis* isolates used for host range characterization showed a high genotypic diversity. Indeed, multilocus sequence typing revealed the presence of 17 different sequence types (STs), including STs commonly encountered in healthcare settings such as ST2 and ST5 [9, 49]. The tested strains included 24 isolates (57.1%) collected from the driveline exit site and 18 (42.9%) from the forehead. Twenty isolates (47.6%) were methicillin-resistant *S epidermis* strains (collected from the driveline [n = 14, 70%] and forehead [n = 6, 30%]), representing antibiotic resistant *S epidermidis* isolated in healthcare settings.

### Patients' Specific Phages Can Kill Bacterial Isolates Infecting Driveline Exit Sites

Phages vB_SepS_BE20 and vB_SepS_BE22 were isolated from 2 respective patients with driveline infections associated with methicillin-resistant *S epidermis* strains (Supplementary Table 2). The first patient (ID1) was infected with an *S epidermidis* ST5 clone. The second patient (ID25) suffered from a polymicrobial driveline infection (DLI) that included *S epidermidis* ST2 and ST731 (a sublineage of ST2 [50]) clones. Both STs include well-known invasive clones isolated worldwide from various healthcare settings [51]. In both cases, patients' specific phages were able to kill the corresponding infective *S epidermidis* strains when assessed in double-layer agar (Figure 2). Importantly, vB_SepS_BE22 from ID25 was the only phage tested that was capable of infecting ST2 and ST731 isolated from that particular patient, providing rationale for a “personalized” phage therapy (Figure 2). As vB_SepS_BE20 is likely a temperate phage-based on the presence of *integrase* or *transcriptional repressor* genes, which limits its usefulness for phage therapy, we focused only on the virulent vB_SepS_BE22 from ID25 in subsequent experiments.

### Phage vB_SepS_BE22 is a Potential Candidate for Personalized Phage Therapy for the Treatment of Biofilm Infections

**Figure 3.**
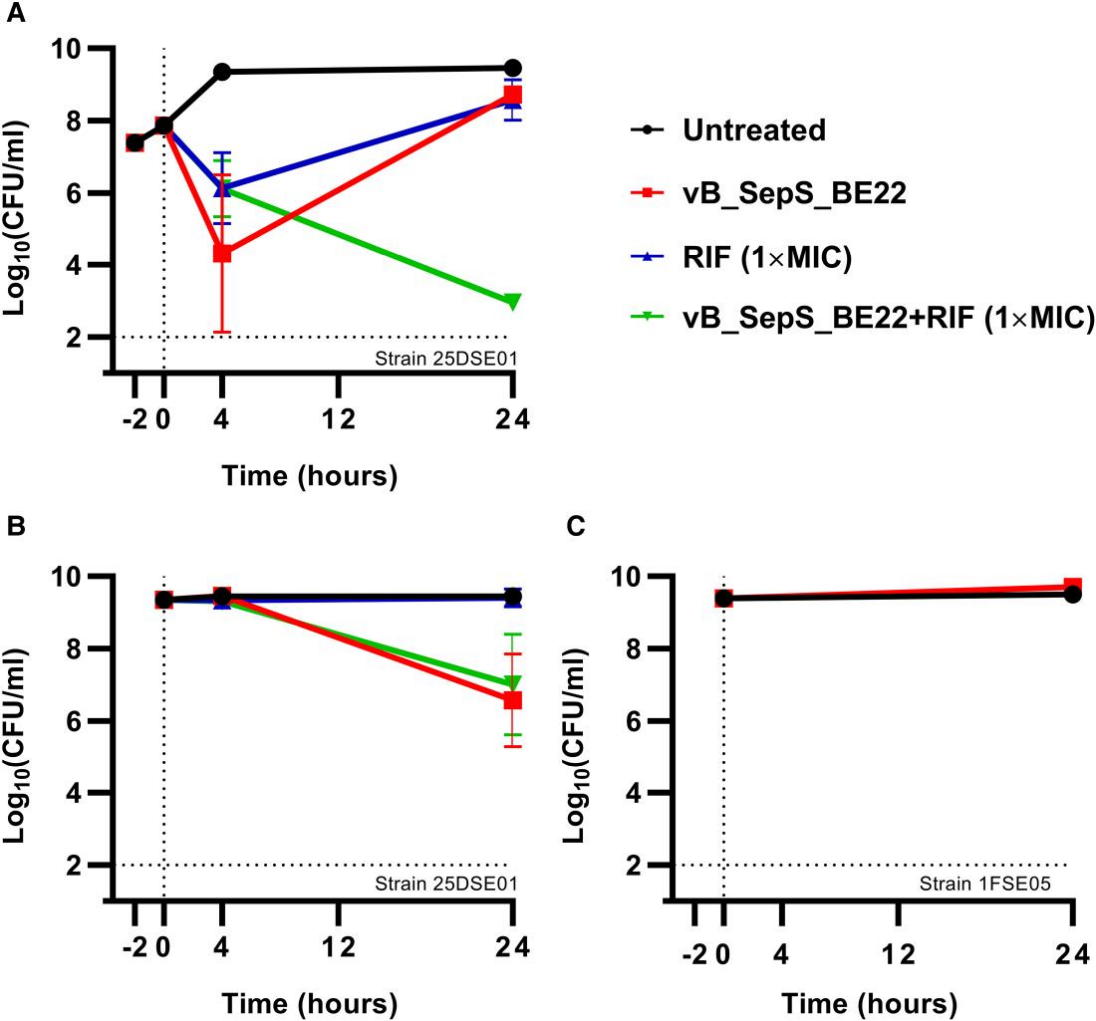
Time-kill assays in planktonic cultures using vB_SepS_BE22, rifampicin, and a combination of both. *S epidermidis* 25DSE01 cells were grown in TSB at 37 °C with shaking (200 rpm) to exponential phase (∼8 × 10^7^ CFU/mL) (*A*) and stationary phase (∼2 × 10^9^ CFU/mL) (*B*). Cultures were then treated with vB_SepS_BE22 (∼2 × 10^7^ PFU), rifampicin (1×MIC [0.008 µg/mL]), and a combination of both. *S epidermidis* 1FSE05 cells were grown in TSB at 37 °C with shaking (200 rpm) to stationary phase (∼2 × 10^9^ CFU/mL) and then treated with vB_SepS_BE22 (∼2 × 10^7^ PFU) (*C*). The limit of detection was set to 10^2^ CFU/mL. Error bars show standard deviations. CFU, colony-forming unit; RIF, rifampicin.

We first assessed the ability of vB_SepS_BE22 to kill *S epidermidis* planktonic cultures in vitro. Experiments were performed using the *S epidermidis* strain 25DSE01, which was isolated from the patient's infected driveline exit site. As adjunctive antibiotic therapy, we included rifampicin because this bactericidal antibiotic is commonly used against staphylococcal biofilms [52, 53]. vB_SepS_BE22 and rifampicin each showed rapid killing after 4 hours when applied alone to exponentially growing cells (Figure 3*A*;  *P* = .0013 and *P* = .0274, respectively, Kruskal-Wallis test followed by Dunn's multiple comparison test). However, bacterial regrowth was observed after 24 hours for both treatments. As expected, the regrowth observed for rifampicin treatment was associated with the emergence of resistance (>125-fold increase in MIC). In contrast, regrowth observed under vB_SepS_BE22 treatment was not due to classical phage resistance, suggesting the involvement of a different mode of phage–host interaction. Combination of vB_SepS_BE22 and rifampicin led to a sustained 6.5-log reduction in the number of survival cells after 24 hours (*P* < .0001) and prevented the emergence of rifampicin-resistant strains (Figure 3*A*).

When applied during the stationary phase, which is classically recalcitrant to both phage infection and the action of antibiotics, vB_SepS_BE22 killed >99% of the cells after 24 hours (*P* = .0038; Kruskal-Wallis test followed by Dunn's multiple comparison test) [54, 55]. In contrast to exponential phase cultures, addition of rifampicin did not improve killing of stationary bacteria compared to vB_SepS_BE22 alone (Figure 3*B*) [54]. To determine if killing of stationary *S epidermidis* was a general feature of vB_SepS_BE22, we tested a second strain from an unrelated patient (strain 1FSE05 from patient ID1). vB_SepS_BE22 did not kill stationary 1FSE05, suggesting this property is not generalizable for *S epidermidis*, and providing further rationale for using vB_SepS_BE22 for the specific purpose of phage therapy for patient ID25's *S epidermidis* infection (Figure 3*C*).

**Figure 4.**
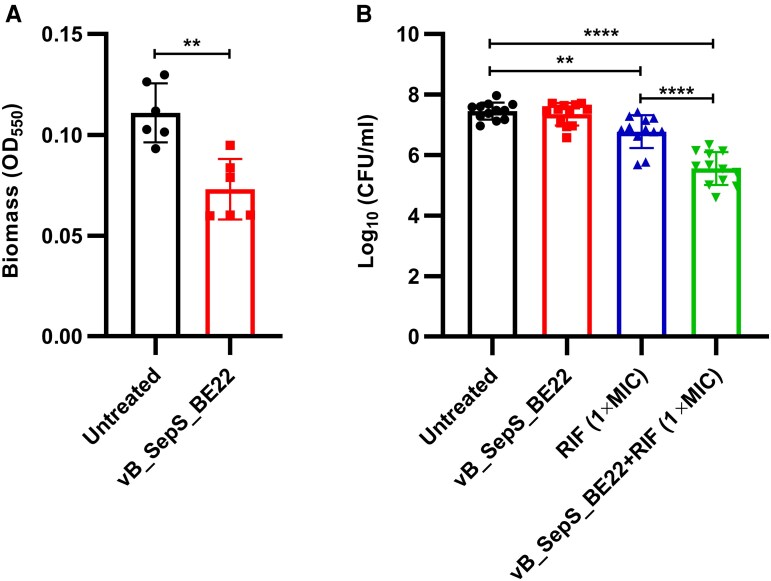
Treatment of biofilms formed in 96-well plates and ex vivo on LVAD driveline sections. *A*, Biofilms were grown for 24 h in 96-well plates, and then treated for 4 h with either phage vB_SepS_BE22 (∼2 × 10^7^ PFU) or TSB (untreated control). Biomass was measured by optical density at 550 nm. Statistical difference was determined using the unpaired *t* test. *B*, Biofilms were allowed to form on LVAD driveline sections for 24 h before treatment with either vB_SepS_BE22 (∼2 × 10^7^ PFU), rifampicin (1×MIC, ie, 0.008 µg/mL), or a combination of vB_SepS_BE22 + rifampicin for an additional 24 h. Viable CFUs were enumerated on TSB agar. The limit of detection was set to 10^2^ CFU/mL. Statistical differences were tested using ordinary 1-way analysis of varianct with Tukey's multiple comparisons test. **P ≤ .01, ****P ≤ .0001. CFU, colony-forming unit; OD, optical density; RIF, rifampicin.

We finally evaluated vB_SepS_BE22 for its capacity to kill *S epidermidis* 25DSE01 biofilms using both a standard crystal violet biofilm staining assay in microtiter plates (Figure 4*A*), and a quantitative ex vivo LVAD driveline biofilm assay (Figure 4*B*). In the crystal violet staining assay, vB_SepS_BE22 lead to a modest biomass reduction after 4 hours (∼1.5-fold, *P* = .0013, unpaired *t* test; Figure 4*A*). When evaluated on biofilms formed on driveline sections, however, vB_SepS_BE22 alone did not achieve a statistically significant reduction in viable CFUs when compared to untreated controls (Figure 4*B*). Rifampicin, a frequently used antibiotic in the context of staphylococcal biofilm infection, produced a modest but statistically significant reduction in CFU for biofilms formed on drivelines (∼0.7-log, *P* = .0034). Combination of vB_SepS_BE22 and rifampicin resulted in 1.9-log reduction in viable CFUs that was statistically superior to each monotherapy (*P* < .0001) (Figure 4*B*).

## DISCUSSION

Treatment strategies that efficiently target biofilms and eradicate difficult-to-kill bacteria are needed to improve clinical outcomes in patients with chronic, biofilm-associated *S epidermidis* infections. In this study, we validated a personalized phage-hunting procedure in patients with LVADs [56]. Phages are natural predators of bacteria and require a host for propagation. They coexisted with their prey for billions of years [57] and are still in a constant evolutionary battle. Indeed, phages have constantly evolved to survive by infecting bacterial populations [58]. Because *S epidermidis* infections can be caused by isolates colonizing the skin surface [59], we reasoned that the skin microbiota would be the most suitable source for the hunting of phages targeting this opportunistic pathogen. Moreover, infections arising from commensal bacteria may result from an imbalance between bacteria and their predatory phages, suggesting that applying these phages therapeutically could help restore this equilibrium.

In our cohort, 3 patients developed an infection associated with *S epidermidis,* and for 2 of them, phages capable of infecting the patient's infective strain were isolated. Most notably, we characterized vB_SepS_BE22, which is a virulent siphovirus isolated from the microbiota of patient ID25. Interestingly, vB_SepS_BE22 was the only phage that killed the infective organism of this patient, suggesting that the skin microbiota can act as reservoir for highly specific phages. Moreover, patient ID25 was harboring the same bacterial strain on the forehead and at the driveline exit site, indicating that the infection might have arisen from the commensal skin microbiota.

The narrow host range of *S epidermidis* mandates a personalized approach for phage therapy, as preformulated cocktails are unlikely to adequately cover the diversity of the species. Phage vB_SepS_BE22 is likely to possess specific properties, which makes it good at killing this specific bacterial strain, including stationary phase cells. Moreover, when combined with rifampicin, antibiotic resistance was suppressed and bacterial burdens in biofilm were reduced.

Previously, we reported the evidence of bacterial transmission between patients [29], and in the current work, we identified the phage vB_SepS_BE20 on the skin of 3 different individuals, suggesting that phages might also be transmitted among patients [60]. However, we could also hypothesize that a common reservoir of *S epidermidis* phages exists among humans, as suggested for *Propionibacterium acnes* phages by Liu et al. [60].

This study brings some advances to the treatment of *S epidermidis* biofilm infections using phages. First, it validated a new phage hunting pipeline that has increased the diversity of isolated phages infecting *S epidermidis*. By combining host-range phenotypic assessments (ie, Figure 2) with phage (Supplementary Figure 3) and host genomic data (Supplementary Table 1), future studies may unravel the molecular underpinnings of the narrow phage susceptibility of the species.

Second, this study is the first of its kind to assess the treatment of staphylococcal infections in laboratory settings simulating LVAD-DLIs in vitro [21]. Although traditional antibiotics, including rifampicin, typically have limited efficacy against biofilms when used alone, our findings suggest that combining phages with antibiotics can enhance the overall treatment efficacy. In this study, rifampicin alone produced a modest reduction in biofilm-associated bacteria, but when combined with phages, the 2 treatments worked synergistically to achieve a more substantial reduction in bacterial burden, consistent with findings from previous studies [61–63]. However, other studies have reported antagonism between phages and rifampicin due to the antibiotic's inhibition of bacterial RNA polymerase [64], suggesting a complex interplay between phages and rifampicin. This highlights the potential for phage–antibiotic combinations to overcome the limitations of antibiotics in treating biofilm-related infections, while also emphasizing the need to thoroughly assess their specific effects in vitro and/or ex vivo before applying them in experimental or clinical settings.

The pipeline does not, however, guarantee that highly virulent phages will be isolated. Indeed, of the 6 unique phages we characterized, half of them were temperate and, as for now, not suitable for therapy. New phage engineering techniques might overcome this limitation [65], but regulatory barriers would still remain for the use of genetically modified phages for therapy. This pipeline requires multiple steps, including phage isolation, purification, and genome sequencing. Despite the potential of generalizing this method to other chronic infection settings, an application for acute infections is currently not feasible because of the insufficient time for designing a personalized treatment. The increased use of artificial intelligence to predict phage–bacteria interactions might reduce the turnaround time for phage selection [66]. Yet, therapeutic phages need to be produced according to good manufacturing practices in order to obtain a safe product, which still remains currently a costly and challenging process [67]. Finally, although our in vitro and ex vivo studies provide a proof-of-concept for personalized phage therapy, further preclinical investigations are necessary to comprehensively evaluate its bactericidal efficacy in vivo before advancing to clinical validation and bedside application.

In summary, our study paves the way for additional therapeutic options in the treatment of biofilm infections. This approach is indeed not restricted to the *S epidermidis* LVAD-DLI setting but may be applied to other indwelling medical devices or implant infections, including those affecting orthopedic prosthesis caused by other problematic bacterial pathogens.

## Supplementary Material

ofaf158_Supplementary_Data

## Supplementary Data

Supplementary materials are available at *Open Forum Infectious Diseases* online. Consisting of data provided by the authors to benefit the reader, the posted materials are not copyedited and are the sole responsibility of the authors, so questions or comments should be addressed to the corresponding author.
