# Supplementary material for: Targeting Chronic Biofilm Infections With Patient-derived Phages: An In Vitro and Ex Vivo Proof-of-concept Study in Patients With Left Ventricular Assist Devices
Source: Open Forum Infect Dis. 2025 Mar 20;12(4):ofaf158. doi: 10.1093/ofid/ofaf158 (PMC11966103; doi:10.1093/ofid/ofaf158)
Supplement: ofaf158_Supplementary_Data [file ofaf158_supplementary_data.zip › OFID-D-24-01486_R1_atl-text.docx]

**Alt text**

**Figure S1**

A flowchart showing the phage hunting process—from sample collection from LVAD patients to phage purification.

**Figure S2**

Electron micrographs of phages isolated from LVAD patients. Four phages belong to the *Siphoviridae* family, and two to the *Herellenviridae* family.

**Figure S3**

Circular genome maps of six novel bacteriophages from LVAD patients. The inner circle shows the GC skew, the second ring shows open reading frames, and the outer rings show similarities to other bacteriophages based on BLAST.
