# Supplementary material for: Targeting Chronic Biofilm Infections With Patient-derived Phages: An In Vitro and Ex Vivo Proof-of-concept Study in Patients With Left Ventricular Assist Devices
Source: Open Forum Infect Dis. 2025 Mar 20;12(4):ofaf158. doi: 10.1093/ofid/ofaf158 (PMC11966103; doi:10.1093/ofid/ofaf158)
Supplement: ofaf158_Supplementary_Data [file ofaf158_supplementary_data.zip › OFID-D-24-01486_R1_Pitton_et_al_supplemental_Wo_Fig.docx]

**SUPPLEMENTAL MATERIAL**

**Targeting chronic biofilm infections with patient-derived phages:
an in vitro and ex vivo proof-of-concept study in patients with left ventricular assist devices.**

Melissa Pitton^1,2^, Luca G. Valente^1,2,3^, Simone Oberhaensli^4,5^, Bülent Gözel^1,6^, Stephan M. Jakob^1^, Parham Sendi^3^, Monika Fürholz^7^, David R. Cameron^1,†^, Yok-Ai Que^1,6,†,#^

^1^Department of Intensive Care Medicine, Inselspital, Bern University Hospital, University of Bern, Bern, Switzerland.

^2^Graduate School for Cellular and Biomedical Sciences (GCB), University of Bern, Bern, Switzerland.

^3^Institute for Infectious Diseases, University of Bern, Bern, Switzerland.

^4^Interfaculty Bioinformatics Unit, University of Bern, Bern Switzerland.

^5^SIB Swiss Institute of Bioinformatics, Lausanne, Switzerland.

^6^Department for BioMedical Research, University of Bern, Bern, Switzerland.

^7^Department of Cardiology, Inselspital, Bern University Hospital, University of Bern, Bern, Switzerland.

† Authors contributed equally

**Table S1. Characteristics of *S. epidermidis* isolates.**

| Isolate ID | Patient ID | Collection site | Date of collection | Sequence type | Accession number |
| --- | --- | --- | --- | --- | --- |
| 1FSE03 | 1 | Forehead | 7-Jul-20 | ST5 | SAMN33711654 |
| 1FSE01 | 1 | Forehead | 7-Jul-20 | ST7 | SAMN33711655 |
| 1FSE05 | 1 | Forehead | 25-Aug-20 | ST5 | SAMN33711656 |
| 1DSE02 | 1 | Driveline | 27-Aug-19 | ST5 | SAMN33711657 |
| 1DSE01 | 1 | Driveline | 27-Aug-19 | ST5 | SAMN32674114 |
| 1DSE05 | 1 | Driveline | 25-Aug-20 | ST297 | SAMN33711658 |
| 4DSE01 | 4 | Driveline | 19-Nov-19 | ST130 | SAMN32674115 |
| 4DSE03 | 4 | Driveline | 25-Feb-20 | ST130 | SAMN33711659 |
| 6DSE01 | 6 | Driveline | 13-Aug-19 | ST731 | SAMN33711660 |
| 6DSE07 | 6 | Driveline | 28-Jul-20 | ST32 | SAMN33711661 |
| 8DSE01 | 8 | Driveline | 13-Aug-19 | ST32 | SAMN32674116 |
| 10DSE01 | 10 | Driveline | 27-Aug-19 | ST5 | SAMN33711662 |
| 16FSE04 | 16 | Forehead | 7-Jul-20 | ST73 | SAMN33711663 |
| 16DSE02 | 16 | Driveline | 27-Jul-21 | ST57 | SAMN33711664 |
| 19DSE01 | 19 | Driveline | 22-Oct-19 | ST731 | SAMN33711665 |
| 20DL | 20 | Driveline | 22-Sep-21 | ST32 | SAMN33711666 |
| 21FSE01 | 21 | Forehead | 2-Sep-20 | ST86 | SAMN33711667 |
| 21FSE04 | 21 | Forehead | 6-Oct-21 | ST7 | SAMN33711668 |
| 21DSE01 | 21 | Driveline | 6-Aug-19 | ST5 | SAMN33711669 |
| 23DSE01 | 23 | Driveline | 12-Nov-19 | ST5 | SAMN33711670 |
| 24FSE01 | 24 | Forehead | 4-Aug-20 | ST35 | SAMN33711671 |
| 24FSE04 | 24 | Forehead | 6-Oct-20 | ST35 | SAMN33711672 |
| 24FSE03 | 24 | Forehead | 6-Oct-20 | ST35 | SAMN33711673 |
| 24DSE01 | 24 | Driveline | 10-Sep-19 | ST5 | SAMN33711674 |
| 24DSE05 | 24 | Driveline | 26-Jan-21 | ST130 | SAMN33711675 |
| 25FSE01 | 25 | Forehead | 21-Jul-19 | ST731 | SAMN33711676 |
| 25FSE09 | 25 | Forehead | 15-Sep-20 | ST631 | SAMN33711677 |
| 25FSE08 | 25 | Forehead | 15-Sep-20 | ST731 | SAMN33711678 |
| 25DSE01 | 25 | Driveline | 15-Sep-20 | ST2 | SAMN33711679 |
| 25DSE06 | 25 | Driveline | 3-Sep-21 | ST2 | SAMN33711680 |
| 27DSE01 | 27 | Driveline | 24-Sep-19 | ST731 | SAMN33711681 |
| 32FSE01 | 32 | Forehead | 4-Aug-20 | ST528 | SAMN33711682 |
| 32FSE03 | 32 | Forehead | 25-Sep-20 | ST208 | SAMN33711683 |
| 32FSE02 | 32 | Forehead | 25-Sep-20 | ST218 | SAMN33711684 |
| 32FSE06 | 32 | Forehead | 25-Sep-20 | ST297 | SAMN33711685 |
| 32FSE07 | 32 | Forehead | 12-Oct-21 | ST528 | SAMN33711686 |
| 32DSE02 | 32 | Driveline | 25-Jun-19 | ST986 | SAMN33711687 |
| 40DSE01 | 40 | Driveline | 22-Jun-21 | NA | SAMN33711688 |
| 41DSE01 | 41 | Driveline | 20-Jul-21 | ST731 | SAMN33711689 |
| 42DSE01 | 42 | Driveline | 20-Jul-21 | ST87 | SAMN33711690 |
| 44DSE01 | 44 | Driveline | 15-Jun-21 | ST32 | SAMN33711691 |
| 47FSE01 | 47 | Forehead | 31-Aug-21 | ST32 | SAMN33711692 |
| F12* | NA | NA | NA | ST60 | NA |

* *S. epidermidis* F12 is the isolate used as host for propagation and for calculating efficiencies of plating for host range determination.

**Table S2. LVAD patients with driveline infections and screened for phage presence.**

| Patient ID | Driveline infection | Driveline infection associated with *S. epidermidis*^†^ | Phage name |
| --- | --- | --- | --- |
| 1 | **+** | **+** | **vB_SepS_BE20** |
| 4 | + | + |  |
| 25 | **+** | **+** | **vB_SepS_BE22** |
| 33 | + | - |  |
| 8 | + | - |  |
| 13 | + | - |  |
| 24 | + | - | vB_SepS_BE21 |
| 16 | + | - | vB_SepM_BE25 |
| 20 | + | - |  |
| 21 | + | - |  |
| 30 | + | - |  |
| 31 | + | - |  |
| 32 | + | - | vB_SepM_BE24 |
| 35 | + | - |  |
| 36 | + | - |  |
| 38 | + | - |  |
| 44 | + | - | vB_SepS_BE26 |
| 46 | + | - | vB_SepS_BE27 |
| 23 | - | - |  |
| 2 | - | - |  |
| 10 | - | - |  |
| 11 | - | - |  |
| 14 | - | - | vB_SepS_BE28 |
| 27 | - | - |  |
| 37 | - | - |  |
| 39 | - | - |  |
| 40 | - | - |  |
| 41 | - | - |  |
| 42 | - | - |  |
| 43 | - | - |  |
| 45 | - | - |  |
| 47 | - | - |  |

^†^ Routine clinical microbiology only performed species identification of all bacteria grown from a swab sample on request. Swab samples growing coagulase-negative staphylococci, *Corynebacterium* spp., and *Cutibacterium* spp. were reported as “skin flora”. Therefore, a driveline infection associated with *S. epidermidis* has been defined as the identification of ≥5 instances where pathogen was reported as “skin flora”, and *S. epidermidis* was detected during or in between these instances.

**SUPPLEMENTAL FIGURE LEGENDS**

**Figure S1. Schematic representation of the phage hunting pipeline. (1)** Samples were collected from the driveline exit site (**1a**) and from the forehead (**1b**) of LVAD patients. **(2)** *S. epidermidis* was identified using selective media and its presence confirmed via matrix-assisted laser desorption/ionization time-of-flight mass spectrometry (MALDI-TOF MS). **(3)** Solution containing putative phages from the patient’s forehead was filtered and concentrated by centrifugation. **(4)** Putative phages were then propagated using the *S. epidermidis* isolate collected from forehead. **(5)** Phage plaques observed on double-layer agar plates were further purified. This figure has been generated in BioRender (<https://BioRender.com/c48k594>).

**Figure S2. Electron micrographs of phages isolated from patients with left ventricular assist devices.** Four newly identified phages belonged to the *Siphoviridae* family (vB_SepS_BE20 **[A]**, vB_SepS_BE21 **[B],** vB_SepS_BE28 **[C],** vB_SepS_BE22 **[D]**)**,** and two to the *Herellenviridae* family (vB_SepM_BE24 **[E]**, vB_SepM_BE25 **[F]**). White lines indicate 100 nm.

**Figure S3. Circular genome representation of six novel bacteriophages isolated from patients with left ventricular assist devices.** For each panel, the internal circle illustrates the GC skew, and the second ring shows open reading frames. The remaining external rings illustrate BLASTn sequence similarities with other bacteriophages. Phages vB_SepS_BE20, vB_SepS_BE21, and vB_SepS_BE28 are related to phages of the *Phietavirus* genus **(A, B, and C)**; vB_SepS_BE22 shows similarity to phages of the *Sextaecvirus* genus **(D)**; and vB_SepM_BE24 and vB_SepM_BE25 are similar to the phages belonging to the *Sepunavirus* genus **(E, F)**. Representations were generated using Proksee (https://proksee.ca/).
