# Supplementary figures and images for: Targeting Chronic Biofilm Infections With Patient-derived Phages: An In Vitro and Ex Vivo Proof-of-concept Study in Patients With Left Ventricular Assist Devices
Source: Open Forum Infect Dis. 2025 Mar 20;12(4):ofaf158. doi: 10.1093/ofid/ofaf158 (PMC11966103; doi:10.1093/ofid/ofaf158)

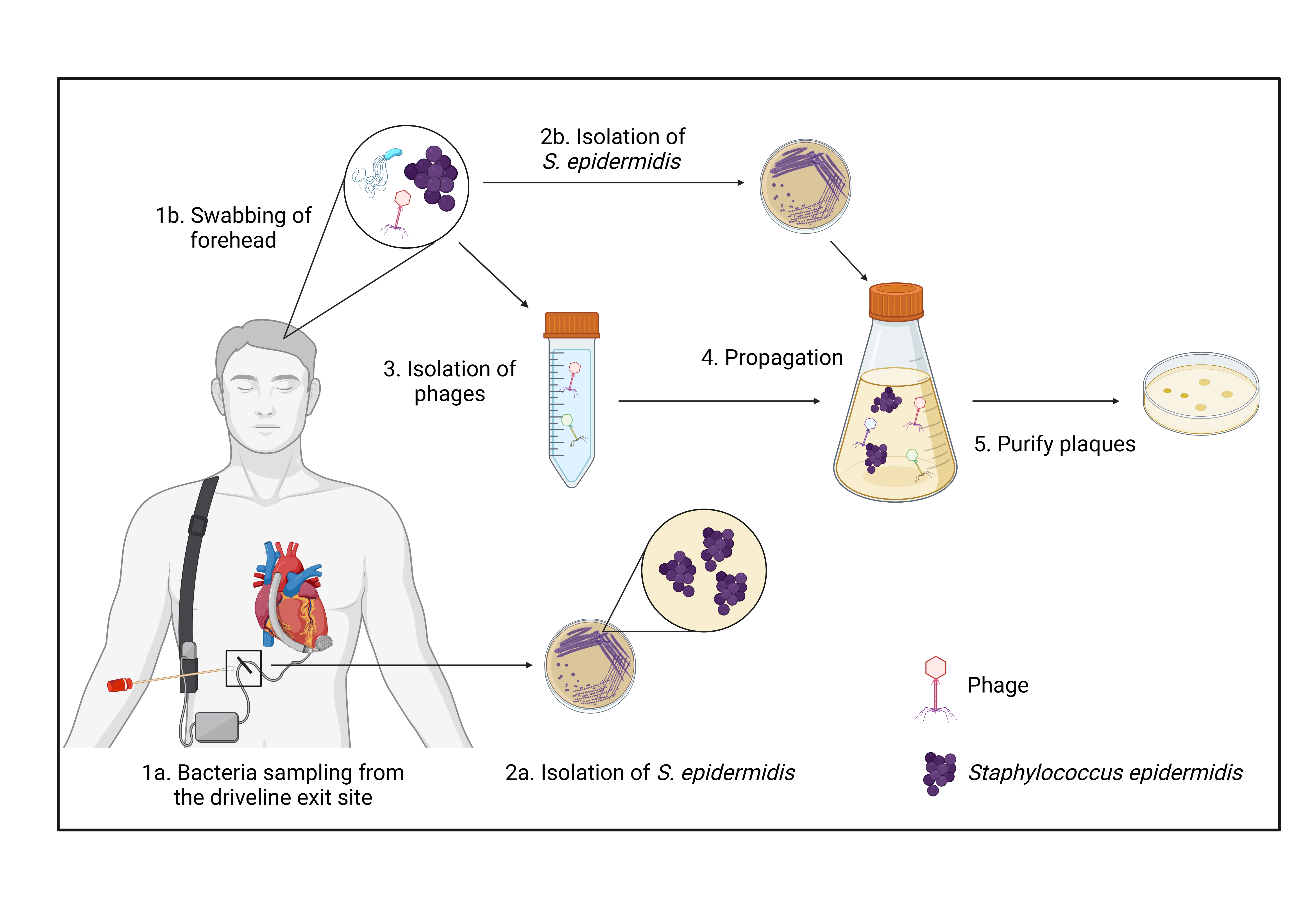

Supplement: ofaf158_Supplementary_Data [file ofaf158_supplementary_data.zip › Figure S1.png]

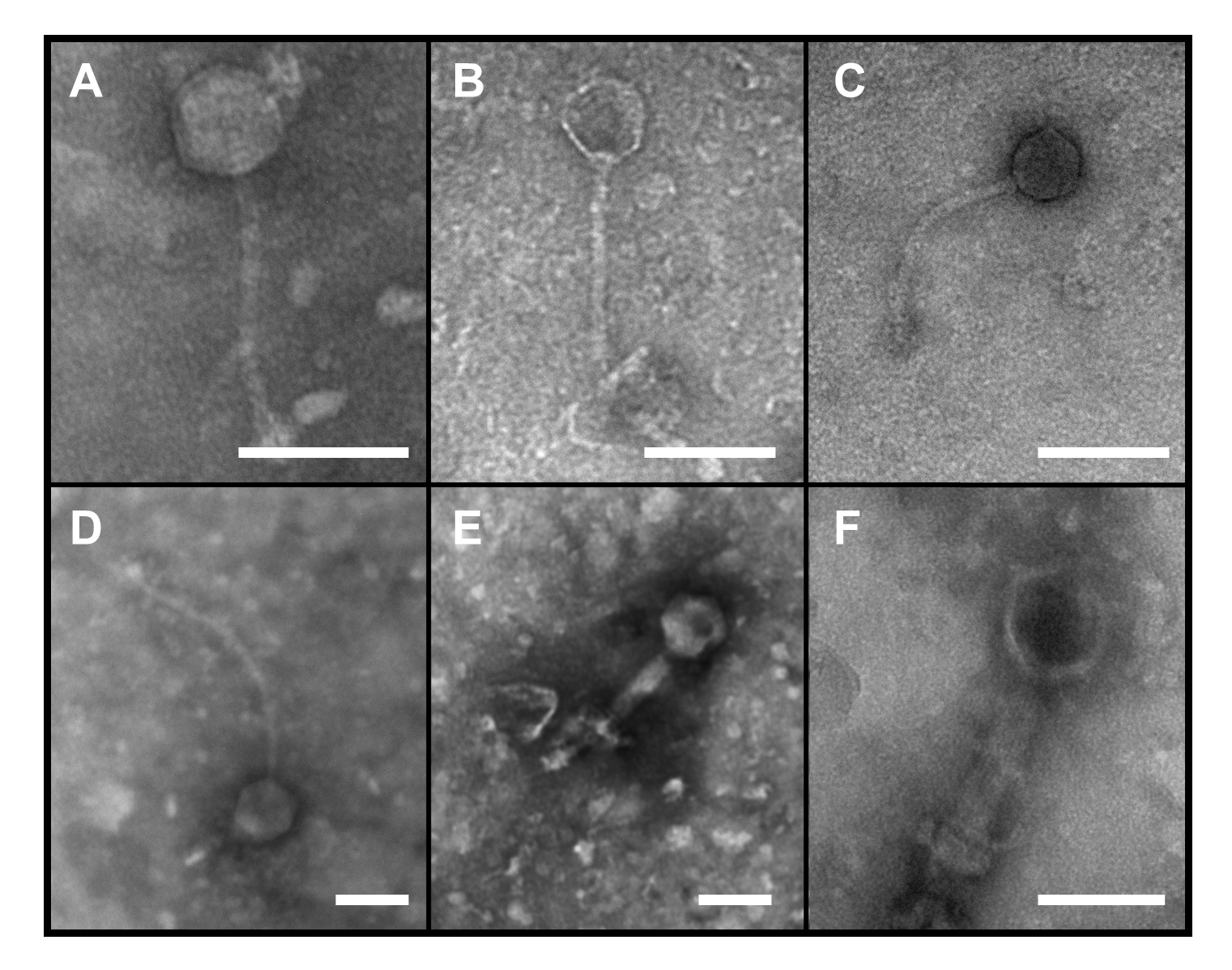

Supplement: ofaf158_Supplementary_Data [file ofaf158_supplementary_data.zip › FigureS2.tif]

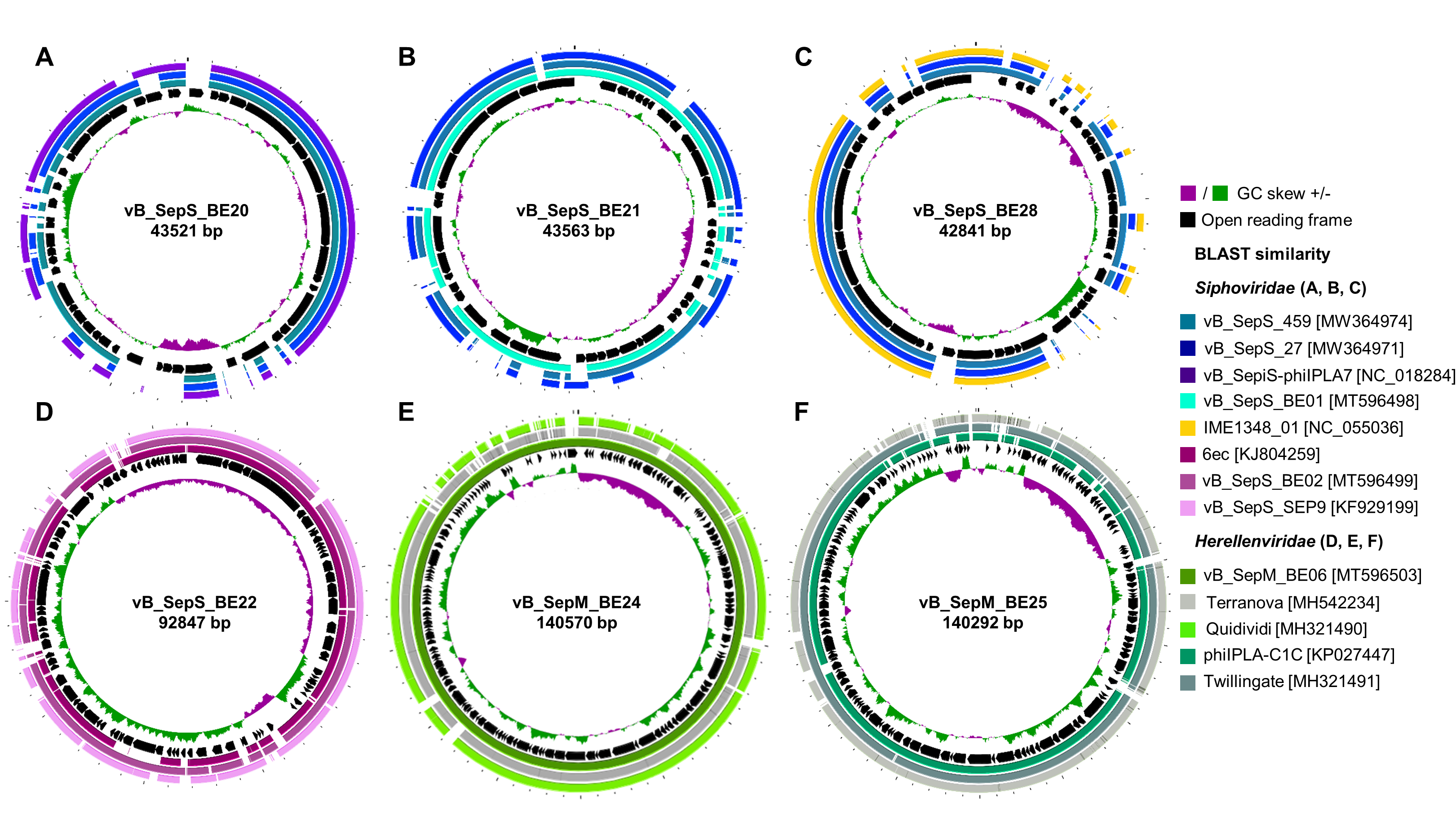

Supplement: ofaf158_Supplementary_Data [file ofaf158_supplementary_data.zip › FigureS3.tif]
